# Dynamic-ETL: a hybrid approach for health data extraction, transformation and loading

**DOI:** 10.1186/s12911-017-0532-3

**Published:** 2017-09-13

**Authors:** Toan C. Ong, Michael G. Kahn, Bethany M. Kwan, Traci Yamashita, Elias Brandt, Patrick Hosokawa, Chris Uhrich, Lisa M. Schilling

**Affiliations:** 1 Departments of Pediatrics, University of Colorado Anschutz Medical Campus, School of Medicine, Building AO1 Room L15-1414, 12631 East 17th Avenue, Mail Stop F563, Aurora, CO 80045 USA; 2 Departments of Family Medicine, University of Colorado Anschutz Medical Campus, School of Medicine, Aurora, CO USA; 3 Departments of Medicine, University of Colorado Anschutz Medical Campus, School of Medicine, Aurora, CO USA; 4 Colorado Clinical and Translational Sciences Institute, University of Colorado Anschutz Medical Campus, School of Medicine, Aurora, CO USA; 5 DARTNet Institute, Aurora, CO USA; 6 OSR Data Corporation, Lincoln, MA USA

**Keywords:** Electronic health records, Extraction, Transformation and loading, Distributed research networks, Data harmonization, Rule-based ETL

## Abstract

**Background:**

Electronic health records (EHRs) contain detailed clinical data stored in proprietary formats with non-standard codes and structures. Participating in multi-site clinical research networks requires EHR data to be restructured and transformed into a common format and standard terminologies, and optimally linked to other data sources. The expertise and scalable solutions needed to transform data to conform to network requirements are beyond the scope of many health care organizations and there is a need for practical tools that lower the barriers of data contribution to clinical research networks.

**Methods:**

We designed and implemented a health data transformation and loading approach, which we refer to as Dynamic ETL (Extraction, Transformation and Loading) (D-ETL), that automates part of the process through use of scalable, reusable and customizable code, while retaining manual aspects of the process that requires knowledge of complex coding syntax. This approach provides the flexibility required for the ETL of heterogeneous data, variations in semantic expertise, and transparency of transformation logic that are essential to implement ETL conventions across clinical research sharing networks. Processing workflows are directed by the ETL specifications guideline, developed by ETL designers with extensive knowledge of the structure and semantics of health data (i.e., “health data domain experts”) and target common data model.

**Results:**

D-ETL was implemented to perform ETL operations that load data from various sources with different database schema structures into the Observational Medical Outcome Partnership (OMOP) common data model. The results showed that ETL rule composition methods and the D-ETL engine offer a scalable solution for health data transformation via automatic query generation to harmonize source datasets.

**Conclusions:**

D-ETL supports a flexible and transparent process to transform and load health data into a target data model. This approach offers a solution that lowers technical barriers that prevent data partners from participating in research data networks, and therefore, promotes the advancement of comparative effectiveness research using secondary electronic health data.

**Electronic supplementary material:**

The online version of this article (10.1186/s12911-017-0532-3) contains supplementary material, which is available to authorized users.

## Background

Clinical data – such as from electronic health records (EHR) - have become key data sources (i.e., as secondary data) for comparative effectiveness research (CER) [1, 2]. The clinical research community has long envisioned using data generated during routine clinical care to explore meaningful health care questions and health policy issues that cannot be addressed by traditional randomized clinical trials [3–7]. Recent developments in observational CER, patient-centered outcomes research (PCOR) study methods, and analytic techniques have improved the ability to infer valid associations from non-randomized observational studies [8–14]. A current objective of multiple major U.S. health data initiatives is to create large CER-supportive data networks by integrating EHR data from multiple sources (i.e., multiple EHRs from multiple health care organizations) and enriching these data with claims data [6, 13–19]. To harmonize data from multiple sources, health data networks transform data from source EHR systems to a common data model (CDM), such as those of the Observational Medical Outcomes Partnership (OMOP), Informatics for Integrating Biology and the Bedside (i2b2), Mini-Sentinel (MS) and the Patient Centered Outcome Research Network (PCORNet) [16, 20–24].

Data harmonization processes are known to consume significant resources, and much prior work has been done to simplify data mappings, shorten data querying time, and improve data quality [25–28]. Common technical challenges of an ETL process are compatibility of the source and target data, scalability of the ETL process, and quality of source data [29–33]. Compatibility challenges occur because local EHR systems often have different data models, vocabularies, terms for data elements, and levels of data granularity. Incompatibility issues may lead to information loss due to the inability of the target data model to translate and store the syntax and semantics of the source data accurately [34]. Scalability is a challenge due to the volume of health data, the need for frequent data refreshes, operational changes in source data systems, and ongoing revisions to target schema definitions and scope. Finally, ensuring data quality as an outcome of the ETL processes is challenging due to the varying quality of source EHR data which is dependent on the source organizations’ EHR implementation and end-user interaction with the system [35]. Another data transformation challenge involves providing solutions for *conflicting* and *duplicate* records. *Conflicting* records are defined as two or more records about the same object (e.g. patient, visit) that share the same identification (e.g. same encounter number) but assert different values for a given fact or observation. On the other hand, *duplicate* records refer to two records that have identical values in all columns except the primary key record identifier. Conflicting and duplicate records are common data problems that can significantly affect the efficiency of an ETL process and output data quality. Current approaches to data transformation are often not flexible or scalable for large initiatives with multiple heterogeneous data sources and highly specified relationships between data elements in the source and target data models [30, 36, 37].

The ETL (Extraction-Transformation-Load) process is a series of operations that allows source data to be syntactically and semantically harmonized to the structure and terminology of the target CDM [38]. The ETL process to support data harmonization typically comprises two sequential phases, each of which is performed by skilled personnel with different expertise. In phase 1, subject matter experts in the source data (e.g. EHR, claims data) identify the appropriate data elements required to populate the target database for extraction and specify the mappings between the source data and target data elements. This step requires knowledge about the structure and semantics of both the source and target data, such as expertise in the local EHR implementation and use, and local terminologies. In phase 2, database programmers implement methods of data transformation and the schema mappings for loading data into the harmonized schema. Transformation is a complex process of data “cleaning” (e.g., data de-duplication, conflict resolution) and standardization (e.g. local terminology mapping) to conform to the target schema format and codes so they can be loaded into the target CDM-specific database. This phase requires manually programming using database-programming languages such as structured query language (SQL). In many cases, these steps are iterated until the transformed data are accepted as complete and correct. These two phases (schema mapping; database programming) must be done separately for each data source, and rarely does a single person have both the source data expertise and database programming skills to perform the tasks in both phases for even a single data source, and especially not for multiple data sources.

The ETL process can be supported by a data integration tool with a graphical user interface (GUI), such as Talend^1^ and Pentaho,^2^ which helps reduce the manual burden of the ETL design process. However, GUI-based tools are often not flexible enough to address complicated requirements of transformation operations such as specific conventions to perform data de-duplication or to perform incremental data load. Also, GUI-based tool often lack transparency of the underlying SQL commands performing the transformation making it difficult to investigate transformation errors.

In this paper, we describe a data transformation approach, referred to as dynamic ETL (D-ETL), that automates part of the process by using scalable, reusable and customizable code, while retaining manual aspects of the process that require complex coding syntax. The contributions of this work include 1) providing a scalable, practical solution for data harmonization in a clinical data research network with heterogeneous source data and 2) lowering the technical barriers for health data domain experts to play the main role in ETL operations by simplifying data transformation process.

## Methods

### Setting and context

SAFTINet (Scalable Architecture for Federated Translational Inquiries Network) is one of three national distributed research networks funded by the Agency for Healthcare Research and Quality (AHRQ) to support broad-scale comparative effectiveness research [21]. In 2010 SAFTINet selected the OMOP version 4 Common Data Model (OMOP v4 CDM) and Terminology as its approach for harmonizing and sharing data across all data partners [32, 39]. Each data-sharing institution that participates in SAFTINet must create and maintain a database that contains their EHR data restructured into a HIPAA-compliant (HIPAA = The Health Insurance Portability and Accountability Act), limited data set that conforms to the OMOP CDM. Clinical data was also integrated with claims data provided by payers, for two safety net organizations, and patient-reported outcomes data collected at the point of care to create the SAFTINet common database.

To ensure compliance with the HIPAA Privacy and Security Rules, SAFTINet restricts protected health information (PHI) data elements to those allowable under the regulatory definition of a limited data set (LDS), which removes direct identifiers, such as name, address and social security number; but includes dates, city/town, state and 3-digit zip codes [40–44]. The D-ETL rules must therefore enforce these HIPAA restrictions as part of the data transformation process.

### D-ETL approach

**Fig. 1:**
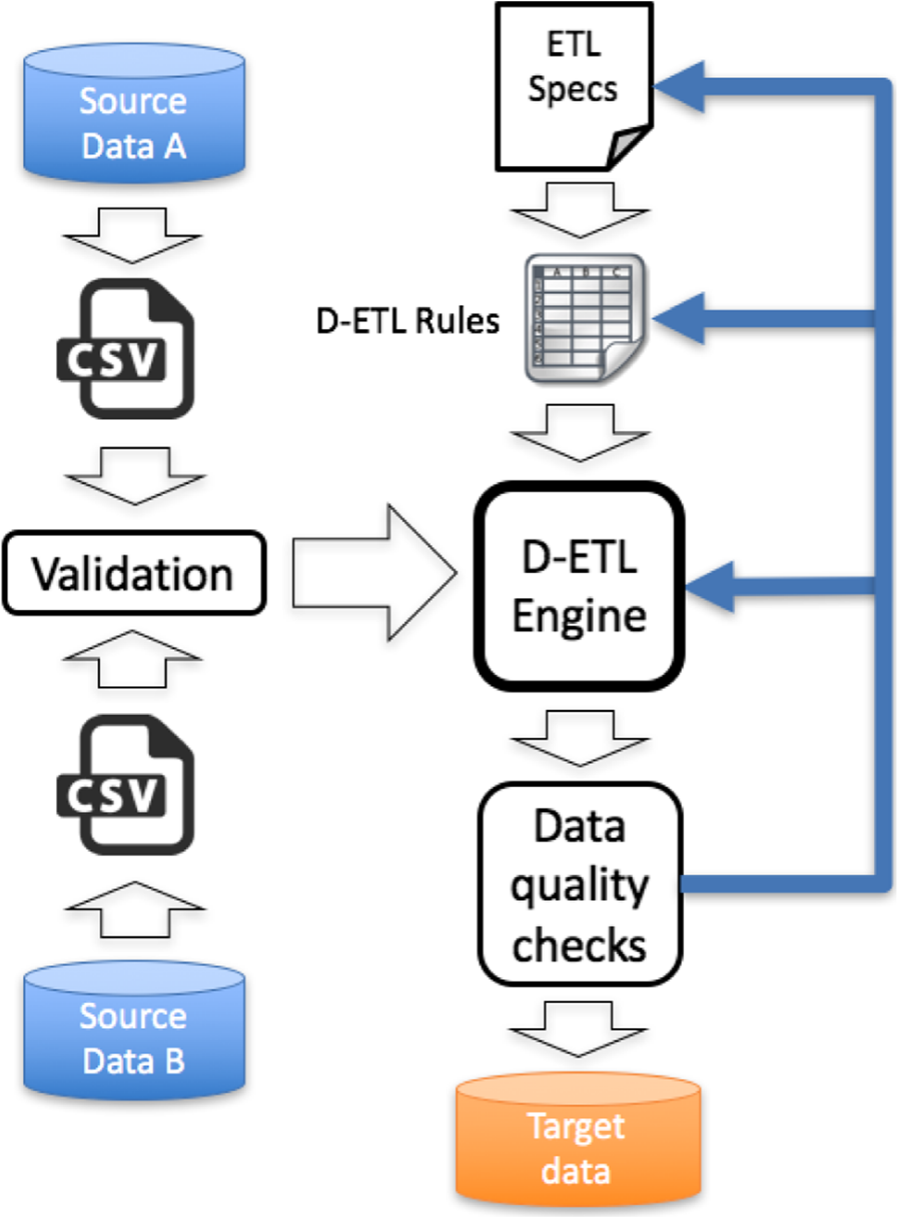
Workflows of D-ETL approach to integration two source datasets

Figure 1 illustrates the workflows in a D-ETL approach to integrate two source datasets. The D-ETL approach is based on four key components:

1. Comprehensive ETL specifications, which are the master plan for the entire ETL process, outlining in narrative text and diagrams the scope of data to be extracted, the target data model, and the format of the input and output data files.
2. D-ETL rules composed in plain text format, which ensures that rules are human readable and therefore easily scrutinized, maintained, shared and reused.
3. An efficient ETL rules engine that generates full SQL statements from ETL rules to transform, conform, and load the data into target tables.
4. These auto-generated SQL statements are accessible by the ETL designers to execute, test and debug the rules thereby supporting an iterative process of validation and debugging.

#### ETL specifications and design

An ETL specifications guidelines (created in a standard word processing application) contains information about the source and target schemas, terminology mappings between data elements and values in the source and target schemas, and definitions and conventions for data in the target schema. The ETL specifications document is created by one or more health data domain experts who have extensive knowledge of the source and target schemas.

#### Data extraction and validation

**Fig. 2:**
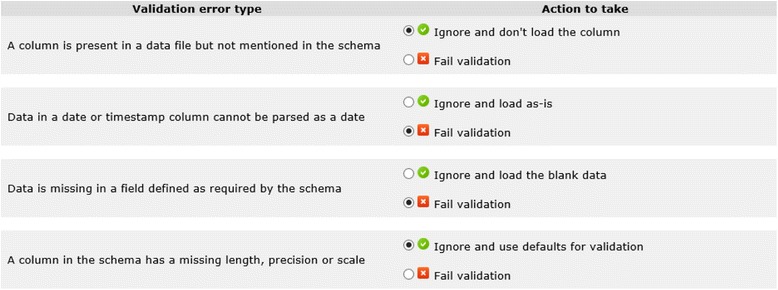
Examples of input data validation rules with loose and strict validation criteria

In the data extraction step, required data elements from the source system are extracted to a temporary data storage from which they are transformed and loaded into the target database. The D-ETL approach uses comma-separated values (CSV) text files for data exchange due to its wide use and acceptability [45]. Extracted data then go through a data validation processes including input data checks for missing data in required fields and orphan foreign key values (e.g. values which are present in a foreign key column but not in a primary key column) checks. In addition, data transformation processes usually have specific assumptions about the value and structure of input data that require validation. Figure 2 shows a list of example validation rules.

#### D-ETL rules

With D-ETL, validated input data are transformed via a set of D-ETL rules. The structure of a D-ETL rule requires basic information about the source and target database (i.e. database, schema, table, field) as well as the data transformation formulae. The structure of the D-ETL rules allows target data to be generated by combining data from *multiple related source tables*. A specific example of a data transformation problem that can be addressed by the ETL rules is the transfer of source data from the source Demographic table and Race field to the target Person table and Race field in the OMOP CDM. Assume that Race values in the source EHR data is coded using the standard Health Level 7 (HL7)^3^ coding system. Since the standard coding system for Race values in OMOP is the Systematized Nomenclature of Medicine (SNOMED),^4^ there must be a terminology mapping operation as part of the ETL process. To address this problem, the D-ETL rule that transforms data in the source Demographic table must reference at least two source tables: The Demographic table and the Source_to_Concept_Map table. The Source_to_Concept_Map table provides the mapping from HL7 value codes for race to SNOMED value codes for race.

A D-ETL rule is a data structure that has 12 attributes and as many rows as needed for complete rule specification. Each rule results in the SQL code that is responsible for the transforming and loading of one or more fields in a *single* target table. Table 1 contains a list of the rule attributes and their descriptions. D-ETL rules are usually composed by the health data domain experts based on the ETL specifications document. D-ETL rules implement the schema mappings prescribed in the ETL specifications document.

**Table 1:** Attribute Type of a D-ETL rule

Attribute	Description	Group
Rule order	Rule identification number. All rows of a rule should have the same rule order	Identification
Rule description	Short description with maximum length of 255 characters to describe the purpose of the rule.	Identification
Target database	Name of target database	Target
Target schema	Name of target schema	Target
Target table	Name of target table	Target
Target column	Name of target column	Target
Map type	Type of row. Possible values: PRIMARY, JOIN, LEFT JOIN, RIGHT JOIN, FULL JOIN, WHERE, VALUE, CUSTOM.	Source
Map order	Identification of row within a rule	Identification
Source database	Name of source database	Source
Source schema	Name of source schema	Source
Source table	Name of source table	Source
Source value	VALUE row: The value used to populate target column JOIN row: join condition WHERE row: where condition	Source

Given its structure, a D-ETL rule can be stored in a CSV formatted file with one column for each attribute. Although D-ETL rules in CSV format can be edited using most text editors available with all major operating systems, experience shows that D-ETL rules can be best composed and maintained in a spreadsheet application. D-ETL rules can be shared easily among ETL teams who have the same source data and target data structure. If multiple data sources are being loaded into a single target dataset, each data source has its own set of D-ETL rules. Table 2 is an example of a D-ETL rule. For simplification, some of the attributes were omitted in the example.

**Table 2:** Example of a D-ETL rule that loads data into the Care_site table in OMOP from a claims-based source CSV file

Rule Order	Rule Description	Target Table	Target Column	Map Type	Map Order	Source Table	Source Value
1	Medical_claims to Care_site	Care_site		PRIMARY	1	Medical_claims	medical_claims.billing_provider_id, medical_claims.place_of_service_code, provider.provider_organization_type
1	Medical_claims to Care_site	Care_site		JOIN	2	provider	medical_claims.billing_provider_id = provider.provider_id
1	Medical_claims to Care_site	Care_site		WHERE	3		provider.provider_organization_type in (‘1’, ‘2’)
1	Medical_claims to Care_site	Care_site	care_site_source_value	VALUE	4		medical_claims.billing_provider_id || ‘-’ ||medical_claims.place_of_service_code||’-’ ||provider.provider_organization_type
1	Medical_claims to Care_site	Care_site	organization_source_value	VALUE	5		NULL
1	Medical_claims to Care_site	Care_site	place_of_service_source_value	VALUE	6		medical_claims.place_of_service_code
1	Medical_claims to Care_site	Care_site	care_site_address_1	VALUE	7		provider.provider_address_first_line
1	Medical_claims to Care_site	Care_site	care_site_address_2	VALUE	8		provider.provider_street
1	Medical_claims to Care_site	Care_site	care_site_city	VALUE	9		provider.provider_city
1	Medical_claims to Care_site	Care_site	care_site_state	VALUE	10		provider.provider_state
1	Medical_claims to Care_site	Care_site	care_site_zip	VALUE	11		provider.provider_zip
1	Medical_claims to Care_site	Care_site	care_site_county	VALUE	12		NULL

Using SQL, the transformation specified in this example D-ETL rule can be done using a pair of SELECT and INSERT statements,^5^ following the syntax below:

**Table:** 

INSERT INTO tableName <fieldList>	
SELECT <Transformed fieldList>	
FROM <tableList>	

The INSERT and SELECT statements above are generated automatically by the D-ETL engine from D-ETL rules. Each component of the rules corresponds to a specific operation of the query. The D-ETL rule engine directly supports the following SQL operations: INSERT, SELECT, SELECT DISTINCT, JOINS (inner join, left outer join, right outer join, full outer join) and WHERE. The D-ETL rule structure takes advantage of both the simplicity of the CSV format and the flexibility of full SQL statements. The D-ETL designer can compose a rule without having extensive knowledge of the formal syntax of SQL, and only requires input from a technical expert for special circumstances (e.g., complex rule debugging). All D-ETL rules used to load a single dataset are contained in one CSV file.

D-ETL rule attributes can be categorized into three functional components: rule specification, output destination and source data. Rule specification attributes include: rule order, rule descriptions and data source ID, a field used to identify specific datasets in case multiple datasets with different rule sets are processed at the same time. A composite key uniquely identifying a rule is formed based on the combination of these three fields. Rule order is the unique identifier of a rule. However, because each rule comprises multiple rows representing that rule’s components, all these rows have the same rule order. Therefore, along with the rule order, each row within a rule is further identified by the map order column. It’s important to note that rule order is unique in one rule set, however, it might not be unique across different rule sets.

The output destination attributes contain information about the target destination (e.g. target database, target schema, target table, target column). A rule can only populate one target table. Not all attributes of the target table must be included in the rule. However, a NULL value is usually use as the source value for non-populated columns.

The source data attributes include the source data information (e.g. source database, source schema, source table, source value). Source data columns not only contain information about the location of the source data but also the data transformation formula.

The example rule in Table 2 is used to populate the target table: Care_site. The rule will be used to generate one SQL statement. The row with PRIMARY map-type identifies the main source table from which the data will be queried, in this example the Medical_claims table. The primary table is a table that has at least one field that is used to populate the primary key of the target table. The map_type column of the first row is always set to “PRIMARY”, indicative of the primary table from which the source data resides. Additional source tables can be joined with the primary table by the join operators with map_type = {JOIN, LEFT JOIN, RIGHT JOIN, FULL JOIN} and the join condition specified in the map_type column. In the example, the source_value column of the PRIMARY row consists of a composite primary key used to populate the primary key of the target table. The primary key includes 3 fields: billing_provider_id and place_of_ service_code from the medical claims table and provider_organization_type from the provider table. The provider table is joined with the medical_claims table in a JOIN row with the JOIN condition specified in the source_value of that same row. An optional row with the WHERE clause can be used with the WHERE condition in the source_value column. For example, the WHERE row defines the WHERE condition that only provider_organization_type of ‘1’ or ‘2’ will be populated to the target table. Note that the “in” operator was used because it is a standard operator that PostgreSQL supports. The next rows, which have VALUE as map_type, contain direct mappings from the source values to the target columns. NULL values must be clearly indicated where target columns cannot be populated. Although all rows in one rule have the same rule_order and rule description, they will have different map_orders. Finally, each value listed in the source_value column must include the source table and source field.

A D-ETL rule can include mappings that have different complexity levels varying from one-to-one mappings to many-to-one mappings. The output of the rule can be filtered by the WHERE clause specified in the map_type column. A WHERE row in a D-ETL rule is not a required component, but when present, each rule can only have one WHERE clause. The source_value column may also contain expressions which can be formulated using native dialect of the DBMS to transform data from the source to target. For example, if PostgreSQL is the DBMS, all PostgreSQL operators and functions are supported. This approach extends the flexibility of the hybrid approach by allowing D-ETL designers to take advantage of all functions supported by the target DBMS. A drawback of this approach is that code translation is needed when the rules are being used in a different DBMS. Therefore, it is good practice to use widely used SQL functions when possible.

#### D-ETL engine

The D-ETL rules are composed in a human-readable format, allowing personnel with limited database programming expertise to compose, read, verify, and maintain them. The D-ETL engine automatically translates these D-ETL rules into complex executable SQL statements to transform and load the data into target tables. During this process, multiple query performance enhancements and data cleaning operations such as common table expressions (CTEs) and data de-duplication are automatically incorporated into the SQL statements.

**Fig. 3:**
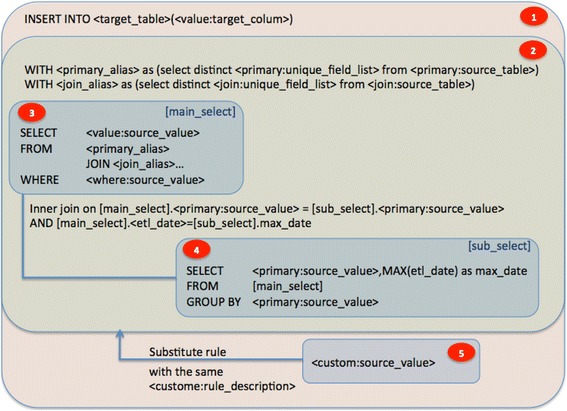
Architecture of the ETL engine

The D-ETL engine comprises five sub-processes that deal with data integration, transformation, de-duplication and loading. In Fig. 3, the numbers in red ovals represent the process used to carry out each D-ETL engine sub-process. In the diagram, variables are enclosed in pointing angle brackets (<variable>) and a query is enclosed in square brackets ([query]). D-ETL rule attributes are identified by the format: <map_type:column_name>. For example, the WHERE condition of a rule can be identified by <where:source_value>. Even though the order of execution is different, for readability, the processes will be enumerated from top to bottom.

In process 1, the D-ETL engine starts by creating the INSERT statement using the values in the target column. In process 2, the data are de-duplicated using sub-queries and CTEs. In process 3, the de-duplicated source data sets are then joined and filtered before being inserted into the target table in process 1. An example solution for conflicting records is to pick the record with latest ETL timestamp out of a group records that share common primary key values. Custom queries are handled in process 5. See Additional file 1 for description of CUSTOM rules and Additional file 2 for detailed description the individual D-ETL engine processes.

#### Testing and debugging

The D-ETL approach facilitates code-level testing and debugging. SQL statements generated by the D-ETL engine are stored separately in the database from individual rules. D-ETL designers can test and debug these statements and review query results directly themselves in an internal testing process. Any syntactic and semantic errors in the statements can be traced back to the components of the rule. This mechanism allows D-ETL designers to understand the error at the SQL statement level and take advantage of the error messaging system of the DBMS, instead of having to go through the indirect error messages provided by a GUI or error log file after a complete ETL process.

In addition, executing the SQL statements directly allows D-ETL designers to iteratively perform trial and error adjustments on the query until the desired transformation is created instead of continuously changing the D-ETL rule. Consequently, only the final change need be recorded in the D-ETL specifications. Being able to review the SQL statements directly allows D-ETL designers to understand the relationship between rule design and the query results, hence, improving the rule design process. A drawback of this testing and debugging process is that it requires direct access to the backend database where the D-ETL statements are stored and might require an advanced knowledge of SQL.

Table 3 summarizes the challenges of the ETL process and the solutions for these challenges enabled by D-ETL.

**Table 3:** Challenges and solutions

Challenges	Solution by D-ETL Approach
Heterogeneity in source data sets	• ETL specifications • Rule-based D-ETL engine • Native SQL code acceptance • Custom rule mechanism
Data extraction interferes with source EHR	• CSV file format
Efficiency	• Integrated D-ETL engine • Query optimization
Duplicate and overlapping data	• Automated data de-duplication and incremental data loading
Data quality	• Input data: Extracted data validation • Output data: Data profiling and visualization
Human expertise	• Explicit rule structure • Effective rule testing and debugging mechanism
Resumption (ability to continue from a point where an error previously occurred)	• Modular ETL process

## Results

### Internal testing and validation

SAFTINet partners successfully implemented D-ETL via a system called ROSITA (Reusable OMOP and SAFTINet Interface Adaptor). The ROSITA software system is operated via a web-based user interface powered by a backend D-ETL engine. The efficiency of D-ETL using ROSITA to process health data is very promising, even in situations where duplicate and overlapping data are present in the source dataset. Table 4 shows the runtime of some D-ETL rules within ROSITA from an internal testing process, loading heath datasets on a CentOS 6 virtual machine with 2 processing cores, 32GB of RAM and 300GB hard drive.

**Table 4:** D-ETL engine performance in ROSITA

Rule number	Number of source tables	Number of records (in all source tables)	Run-time (in seconds)
1	1	21,565	1.1
2	2	851,706	30.3
3	2	1,910,513	12.0
4	2	1,324,860	13.1
5	3	1,987,582	15.3
6	3	2,007,661	30.1

### Practical, scalable and feasible implementation of D-ETL in ROSITA

Using ROSITA, SAFTINet partners in all three states were able to load clinical data and transform the data into the OMOP v4 CDM and in two of these states, where claims data was available, partners were able to load and link clinical and claims data, prior to completing the data transformation to OMOP. Two partners with well-staffed, sophisticated informatics departments implemented ROSITA within their own environment, mapping directly from their own EHR or electronic data warehouse (EDW) to OMOP. The other ten partners used intermediaries who were health data domain experts but not advanced SQL database programmers to transform their EHR data to an intermediary data model; they then applied D-ETL within ROSITA to transform data from the intermediary model to OMOP. The four resulting SAFTINet-affiliated ROSITA instances currently contain records for 1,616,868 patient lives. These ROSITA systems have also been used to capture results of over 8000 patient-reported outcomes measures, which are being used in SAFTINet CER studies [46, 47].

A site-specific ETL specifications guideline is used by each data partner to both guide and document their extracted source data choices and intended target locations. Source data is extracted and transferred using CSV files. In practice, the CSV format is a ubiquitous and flexible temporary storage for D-ETL. Extracting data to CSV files allowed the data extraction process to be separated from the more difficult data transforming and loading processes. Separating data extraction from data transformation eliminated the need for an active network connection to the source data every time a new transformation task was performed. In addition, extracting the source data into a temporary storage system, without directly connecting to the source database, enabled controlled access to the exported data created by the data owners.

Data quality validations on the extracted dataset were important to ensure the success of the subsequent steps. Data validation usually occurred immediately after the data extraction step for quick identification of any problems with the extracted data and expedited re-extraction. Experience showed that it is very difficult to produce perfectly extracted data sets that would be accepted by the ETL process on the first try. For that reason, it was important to learn from the errors and incorporate them back into the data extraction conventions document for future extractions. Data in CSV files can be validated directly or imported as-is into a DBMS, ideally the same DBMS where the transformation will occur. Table 5 includes a list of data validation rules performed to ensure the quality of the data is appropriate for ETL processes.

**Table 5:** Examples of extracted data validations

Validation Rule	Type of error	Description
Data in a date or timestamp column cannot be parsed as a date	Error	Invalid date data will fail date operators and functions
Data is missing in a field defined as required by the schema	Error	Missing data in required fields will violate database constraints of target schema
A column in the schema has a missing length, precision or scale	Warning	Default length, precision or scale can be used
Data in a numeric or decimal column is not a number	Error	Invalid numeric data will fail numeric operators and functions
Data is too long for text or varchar field	Error	Data loss will occur if long text values are truncated to meet length requirement

In Table 5, if the type of error is “Error”, the data fail the validation rule and have to be extracted again after the problem is fixed. The data element that causes the error and its exact location in the dataset must be provided. To protect sensitive data from being exposed, only the line number of the data record in the data file is displayed. If the type of error is “Warning”, the data will not fail the validation rule. Instead, there will be a warning message that provides information about the data. The decision to deal with the issue is optional. The list of validation rules is based on the anticipation of the requirements of the data transformation process into the target data model. The type of error can be classified based on the impact of the error and the expected amount of information loss.

In SAFTINet, a health data domain expert, who had limited expertise about SQL or the DBMS, created a separate D-ETL rule set for each partner’s OMOP CDM instantiation. Occasionally, the domain expert required assistance from technical personnel for complex DBMS functions. Technical assistance was also needed in case of non-obvious data output discrepancies. Over time, the number of technical assistances might diminish as the experience of the domain expert In many cases, the health data domain expert was able to compose, load and debug the rules. Via the operations of ROSITA, we found that D-ETL is very effective in rule testing and debugging. The health data domain expert was able to effectively track down the source of the error by executing individual rules.

Extending beyond SAFTINet, ROSITA has been used by the DARTNet Institute (DARTNet) to successfully transform data for more than 7 million patients into the OMOP CDM for various CER, quality improvement and interventional research activities. DARTNet uses ROSITA in a different way than SAFTINet partners; data contributors send fully identified data to DARTNet personnel (under HIPAA BAAs) who then perform transformations centrally (i.e., in a non-distributed fashion).

## Discussion

In this project, we designed and implemented a novel hybrid rule-based ETL approach called Dynamic-ETL (D-ETL). Implementation in practice shows that D-ETL and its implementation in ROSITA is viable and successful approach to the structural and sematic harmonization of health data in large health data sharing networks containing heterogeneous data partners, such as SAFTINet and DARTNet. D-ETL allows health data domain experts with limited SQL expertise to be involved in all phases, namely ETL specifications, rule design, test and debugging rules, and only requires expert technical assistance in special cases. D-ETL promotes a rule structure that accommodates both straightforward and complex ETL operations and support ETL transparency and encourages D-ETL rule-sharing. The ETL rule engine also incorporates the mechanisms that deal with conflicting and duplicate data. Using readily available hardware, the implemented D-ETL system shows acceptable performance results loading real health data.

The accuracy and reliability of the D-ETL rules and the D-ETL engine rely on the accuracy and reliability of content of the D-ETL rules. Additional technical performance metrics to be examined in a larger scale implementation would include aspects of improved data quality, including accuracy (e.g., fewer errors in mapping) and completeness (e.g., fewer missing data points) [48]. Other key factors in adoption and use of D-ETL include perceived usability and usefulness and resources and time required to implement the system, which can be assessed using surveys and in-depth interviews with users [49]. In this initial development and small scale implementation work, clinical partner users reported the domain experts following the D-ETL approach experienced a learning curve and increased efficiency following the first use. A better understanding of the efforts related to D-ETL rule composition and debugging, and level of involvement of database technicians in comparison with other tools or approaches will further validate the effectiveness and relative efficiency of D-ETL in different contexts. It is important to note the performance of an ETL operation with healthcare data depends on many interrelated factors such as familiarity with source data model and the complexity of the terminology mapping process which are external to the operations of the D-ETL engine.

Despite its advantages, D-ETL has several limitations. First, although expertise in query writing is not required, certain SQL coding skill is needed for the health data domain expert who is involved in the ETL process. Knowledge about the operators and functions of the DBMS is needed for the rule creation. Second, since the ETL rules are composed in third party software such as Excel, no real time syntactical error checking is available. The rule composer will not know about syntactical errors (i.e. incorrect column name) until the SQL statement is generated. Third, the testing and debugging process requires direct access to the rule database and extract data dataset, which might not be available to the rule composer due to database security access limitations.

Future directions of D-ETL focus on addressing some of the limitations and improving the efficiency of the rule designing process. First, the emergence of semi-automatic schema mapping methods supports the automation of D-ETL rules composition [50]. The involvement of the health data domain expert can then focus more on correcting the mapping results and ensuring data quality. Second, an automatic rule validation mechanism that checks for basic syntactical errors would improve the efficiency of the ETL rule creation process. To be feasible, a user-friendly rule editor with intuitive user interface has to be developed. Third, the expressions in the ETL rules must be in the language of the local DBMS. For rules to be used and reused across different DBMSs, a rule conversion tool that automatically translates operators and functions from one SQL dialect into another is needed. Open source tools, such as SQL Renderer from the OHDSI community,^6^ could be a potential solution to this problem. Finally, even though rules are composed in plain text format, a graphical presentation of the structure of the rule will improve ETL rule maintenance and help ETL team members understand complex rules created by others.

## Conclusion

Data harmonization is an important step towards data interoperability which supports the progression of comparative effectiveness research. Data harmonization can be accomplished by incorporating data standards, the knowledge of domain experts and effective and efficient ETL processes and tools. In this work, we propose a dynamic data ETL approach to lower the technical barriers encountered during execution of ETL processes. Our approach has been implemented and deployed to load clinical and claims data from source electronic health record systems into the OMOP common data model. This is an important step forwards to making high quality data available for clinical quality improvement and biomedical research.

## Additional files

Additional file 1: Description of the custom D-ETL rules. (DOCX 13 kb)

Additional file 2: Description of the D-ETL engine. (DOCX 21 kb)

## Abbreviations

AHRQ: Agency for healthcare research and quality
BAA: Business associate agreement
CDM: Common data model
CER: Comparative effectiveness research
CSV: comma-separated values
DARTNet: Distributed ambulatory research in therapeutics network
DBMS: Database management system
D-ETL: Dynamic extraction, transformation and load
EDW: Electronic data warehouse
EHRs: Electronic health records
ETL: Extraction, transformation and load
GUI: Graphical user interface
HIPAA: Health insurance portability and accountability act
HL7: Health level 7
I2b2: Informatics for integrating biology and the bedside
ICD-9CM: International classification of diseases, ninth revision, clinical modification
LDS: Limited data set
MS: Mini-Sentinel
OHDSI: Observational health data sciences and informatics
OMOP: Observational medical outcome partnership
PCOR: Patient-centered outcomes research
PCORnet: Patient centered outcome research network
PHI: Protected health information
ROSITA: Reusable OMOP and SAFTINet Interface Adaptor
SAFTINet: Scalable architecture for federated translational inquiries network
SNOMED: Systematized nomenclature of medicine
SQL: Structured query language

## Footnotes

1
https://www.talend.com/

2
http://www.pentaho.com/

3
http://www.hl7.org/

4
https://www.nlm.nih.gov/research/umls/Snomed/snomed_main.html

5
http://www.w3schools.com/sql/sql_insert.asp

6
https://github.com/OHDSI/SqlRender
