# Supplementary material for: Dynamic-ETL: a hybrid approach for health data extraction, transformation and loading
Source: BMC Med Inform Decis Mak. 2017 Sep 13;17:134. doi: 10.1186/s12911-017-0532-3 (PMC5598056; doi:10.1186/s12911-017-0532-3)
Supplement: Supplementary file 1 — Description of the custom D-ETL rules. (DOCX 13 kb) [file 12911_2017_532_MOESM1_ESM.docx]

**Additional file 1 – Description of the custom D-ETL Rules**

D-ETL also provides a custom rule solution for rules that do not fit the rule structure above. A custom rule allows the SELECT statement to be a user-defined query that can be any SELECT query supported by the DBMS. It is required that the INSERT statement and SELECT statement of a custom query are constructed separately using two different rules: INSERT rule and SELECT rule. The INSERT rule will be defined using a normal rule structure. In the INSERT rule, only cells in the target columns are required to be filled and cells in the columns identifying the source must be empty. The SELECT rule is a one-row rule that should have a unique rule order with the same rule description as the INSERT rule. Also, the map_type column of the SELECT rule should be set to “CUSTOM”. The source_value column of the SELECT rule contains an SQL statement that transforms source data that is loaded by the INSERT rules. Table 1 contains an example of the one-row CUSTOM rule. The Custom rule mechanism gives the rule composer the full power of the programming language’s transformation and goes beyond the limit set by the normal rule architecture such as data aggregation, functions, sub-queries or common table expressions (CTEs). The custom query will be used instead of the SELECT statement of the rule generated by the INSERT rule.

Table 1 - Example of the one-row CUSTOM rule

| Rule Order | Rule Description | Target Table | Target Column | Map Type | Map Order | Source Table | Source Value |
| --- | --- | --- | --- | --- | --- | --- | --- |
| 2 | Medical_claims to Care_site |  |  | CUSTOM | 1 |  | SELECT … FROM … |
