# Supplementary material for: Dynamic-ETL: a hybrid approach for health data extraction, transformation and loading
Source: BMC Med Inform Decis Mak. 2017 Sep 13;17:134. doi: 10.1186/s12911-017-0532-3 (PMC5598056; doi:10.1186/s12911-017-0532-3)
Supplement: Supplementary file 2 — Description of the D-ETL engine. (DOCX 21 kb) [file 12911_2017_532_MOESM2_ESM.docx]

**Additional file 2 – Description of the ETL Engine**

**d-etl engine processes**

Process 1-5:

Process 1: Generating INSERT statement

| For Each rule $r$  Set insert_statement to “INSERT INTO ”  <primary:target_table>    For each VALUE row  Add <value:target_column> to insert_statement  End For  End For |
| --- |

Description: The objective of Process 1 is to construct the INSERT statement that loads the transformed data into the target table. This is usually the last step in the execution order of an ETL query because before loading, the data have to be transformed, filtered and cleansed. There can be only one INSERT statement in each ETL query. Note that the name of the target table should be placed in the target table column of the PRIMARY row. In addition, each ETL rule can only populate one target table. The process depends entirely on the target column of each rule to generate the field list to place next to the insert statement. Only the target column in the VALUE rows will be used to compose the field list. The fields that are in the target table but are not present in one of the VALUE rows will be populated with a NULL value. Therefore, the D-ETL designer should make sure that required fields such as primary keys in the target table are properly declared in the rule. It is a good practice to include all fields of the target table in the rule and use NULL in the source_value column for unpopulated fields (example: map order #5 and #12 in **Error! Reference source not found.** in APPENDIX A). As an example, the INSERT statement below was generated for the Care_site rule:

| INSERT INTO care_site(care_site_source_value, organization_source_value, place_of_service_source_value, care_site_address_1, care_site_address_2, care_site_city, care_site_state, care_site_zip, care_site_county) |
| --- |

Process 2 – Generate Common Table Expression (CTE) statements

| For Each rule $r$  -- De-duplicate the data in source tables  Set <$s_{id}$> to data source ID number of rule $r$  For Each source table $t$  Set distinct field list <$dfl$> to empty  For Each PRIMARY, JOIN, WHERE, and VALUE rows  For Each field $f$ in the source_value column  If $f\in t$AND $f\notin$ <$dfl$>  <$dfl$> = <$dfl$>$\bigcup f$  End If  End For  <$dfl$> = <$dfl$> $\bigcup$ <data_source_id> $\bigcup$ <etl_date>  Create a SELECT DISTINCT statement selects <$dfl$> list from $t$WHERE data_source_id = <$s_{id}$>  Assign an alias to each SELECT DISTINCT statement  End For  Create and CTE WITH clause from the SELECT DISTINCT statements  End For |
| --- |

Description: Process 2 is the process to de-duplicate source data. De-duplication is important to data loading because duplicate data will increase the size of the query result exponentially and therefore significantly affects query performance. De-duplication is also important to improve data quality of the outcome dataset. For example, a dataset with duplicate data will result in incorrect row counts and incorrect interpretation of the data. The de-duplicating processes have to be performed dynamically for each rule because different rules use different sets of source columns to populate the target table. In the example in Table **1**, even though these are not duplicate records, if a rule uses only four columns PatID, First Name, Last Name and DOB to obtain date of birth of a patient, there will be duplicates in the four columns being used.

Table 1 - Duplicate data example

| PatID | First_Name | Last_Name | DOB | Provider_ID |
| --- | --- | --- | --- | --- |
| 10001 | John | Smiths | 11/03/2001 | P02342 |
| 10001 | John | Smiths | 11/03/2001 | P12384 |

The de-duplication process can be performed using a sub-query for each source table. For each source table (PRIMARY and JOIN) of a rule, a distinct list of its fields is extracted from the source_value column of all rows of that rule. Therefore, only fields that appear in the source_values column of a rule will be included in the sub-query of that table. The DISTINCT clause is used in each SELECT statement to perform the de-duplication. It is recommended to use CTEs by using the WITH clause which has better readability than inline sub-queries. A unique alias should be assigned to each sub-query. If data from multiple data sources are being loaded, it is important to limit to only the data source that these rules are applied to. The sub-queries within the WITH clause is the first component of the whole SQL statement that is executed. Below is the WITH clause generated from the Care_site rule:

| WITH  a1 as SELECT DISTINCT billing_provider_id, place_of_service code FROM medical_claims;  a2 as SELECT DISTINCT provider_organization_type, provider_id, provider_organization_type, provider_address_first_line, provider_street, provider_city, provider_state, provider_zip; |
| --- |

Process 3 – Generating the main_select

| For Each rule $r$  -- SELECT statement  Set <main_select> to “SELECT DISTINCT ”  For Each VALUE row  <main_select> = <main_select> $\bigcup$ <value:source_value>  End For  -- FROM  Set <main_from> to “FROM ”  Set PRIMARY alias for <main_select>  For Each JOIN row  <main_form> = <main_form> $\bigcup$ <join:map_type> $\bigcup$ <sub_query_alias>  ON <join:source_value>  End For  -- WHERE  Set <main_where> to “WHERE ”  For Each WHERE row  <main_where> = <main_where> $\bigcup$<where:source_value>  End For  <main_select> = <main_select> $\bigcup$ <main_from> $\bigcup$ <main_where>  End For |
| --- |

Description: Process 3 describes the process of generating the components, namely SELECT, FROM and WHERE of the main query. While SELECT and FROM are two required components of a query, WHERE is optional. Therefore, an ETL rule might not have a WHERE row. The SELECT statement not only projects data columns from the source table but also performs data transformation. Similar to the creation of the INSERT statement in process 1, the process to create the SELECT statement also scans the VALUE rows but focuses on the source_value column instead. Since both INSERT and SELECT statements are generated from the same set of rows, the number and the order of appearance of the columns in these two statements are the same. Note that the result of the main_select also need to be de-duplicated.

The FROM clause identifies the source tables of the expressions in the SELECT statement. Because the source tables were de-duplicated by the CTEs, aliases should be used instead of actual table names. Since different source tables might have similar column names, the columns in the SELECT statement have to be preceded by the alias in the FROM clause. The FROM clause has to start with the source table in the PRIMARY row, then joined to other tables in the JOIN rows. The JOIN type is set in the map_type column of JOIN rows. The join condition can be found in the source_value of JOIN rows. The WHERE condition can be found in the source_value column of WHERE rows. Below is the main select of the Care_site rule:

| SELECT a1.medical_claims.billing_provider_id \|\| '-' \|\|a1.medical_claims.place_of_service_code\|\|'-' \|\|a2.provider.provider_organization_type, NULL, a1.place_of_service_code, a2.provider_address_first_line, a2.provider.provider_street, a2.provider_city, a3.provider_state, a3.provider_zip  FROM a1 JOIN a2 on a1.billing_provider_id = a2.provider_id  WHERE a2.provider_organization_type in ('1', '2') |
| --- |

Process 4 – Processing incremental data load

| For Each rule $r$  Set <main_select> to the select statement generated by process 3  Set <key_fields> to source value of PRIMARY row  Set <most_recent_select> to  SELECT key_fields, MAX(etl_date)  FROM main_select  GROUP BY key_fields  <main_select> = <main_select> JOIN <most_recent_select> ON  main_select.elt_date = most_recent_select.etl_date AND main_select.key_fields = most_recent_select.key_fields  End For |
| --- |

Description: Process 4 describes the process that deals with overlapping data. Overlapping data are data records that have common values in key fields. The key fields identified by the domain expert are contained in the source_value of the PRIMARY row. For example, in the Care_site rule, the key fields are billing_provider_id, medical_claims.place_of_service_code, and provider_organization_type. The convention to deal with overlapping data is to pick the record that has the latest information, which is identified by the ETL timestamp. ETL timestamp is recorded as the data is loaded into the temporary database. To find the most recent record, a sub-query is needed to find the latest ETL timestamp for each set of key fields. The sub query will be joined back to the main query to keep only records with the latest ETL timestamp in the main query. Note that in case of incremental data loading, the overlapping data processing has to happen before the load to the target schema. For each incremental data patch, both existing and incremental data have to be loaded again to the target data as one dataset. Below is an example of the sub-query that finds the latest ETL timestamp for each instance of the key fields in the Care_site rule.

| SELECT a1.medical_claims.billing_provider_id \|\| '-' \|\|a1.medical_claims.place_of_service_code\|\|'-' \|\|a2.provider.provider_organization_type, Max(ETL_TimeStamp) FROM a1 JOIN a2 on a1.billing_provider_id = a2.provider_id WHERE a2.provider_organization_type in ('1', '2')  GROUP BY a1.medical_claims.billing_provider_id \|\| '-' \|\|a1.medical_claims.place_of_service_code\|\|'-' \|\|a2.provider.provider_organization_type |
| --- |

Process 5 – The custom rule mechanism

| For Each rule r  If <primary:map_type> = CUSTOM  Set <custom_description> = <primary:rule_description>  Set <custom_source_id> = <primary:data_source_id>  Set <custom_select> = <primary:source_value>    REPLACE <main_select> with <custom_select>  WHERE <main_description> = <custom_description  AND main_data_source_id = custom_source_id  End If  End For |
| --- |

A CUSTOM rule is a one-row rule with map_type of CUSTOM and source_value as the full Select statement. A CUSTOM rule must be preceded by an empty INSERT rule whose select statement will be replaced by the source_value of the custom rule. While the CUSTOM rule can have a different rule order, it must have the same rule description and data source identification as the corresponding INSERT rule. A general remark for CUSTOM rules is that although the CUSTOM rule mechanism greatly improves the flexibility of the ETL rule engine, it allows the execution of unstructured SELECT statements, which are error prone. An example of a common error could be an unmatched number of fields in the SELECT statement and the INSERT statement.
